# Phytoremediation capacity of tomato against Sb (III) focusing on the AsA/GSH cycle

**DOI:** 10.3389/fpls.2026.1776021

**Published:** 2026-05-28

**Authors:** Inmaculada Garrido, Francisco Espinosa, Francisco L. Espinosa-Vellarino, Carmen Gloria Relinque, Alfonso Ortega

**Affiliations:** 1 Research Group of Physiology, Cellular and Molecular Biology of Plants, University of Extremadura, Badajoz, Spain; 2 Instituto Universitario de Investigacion de Recursos Agrarios (INURA), University of Extremadura, Badajoz, Spain

**Keywords:** antimony, ascorbate, glutathione, proline, tomato

## Abstract

Antimony (Sb) is a toxic metalloid whose presence in the environment has increased in recent years due to anthropogenic activities. In soils, this element may occur as the pentavalent ion Sb(V) (antimonate) or the trivalent ion Sb(III). The latter is more abundant in nature and more harmful to living organisms. In this study, the effects of Sb(III) on a model plant of major agronomic interest – tomato – are investigated in order to elucidate which compounds or metabolic pathways may be key in the plant response to this stressor. In order to achieve this objective, tomato seedlings were cultivated hydroponically and exposed to varying concentrations of Sb(III). This approach ensured optimal availability of the metalloid to the plants. The results of the present study demonstrate that exposure to Sb(III) inhibits plant growth and triggers a range of defence mechanisms, among which proline, phytochelatins (PCs) and enzymes of the AsA/GSH cycle play a prominent role in xenobiotic detoxification. In particular, ascorbate peroxidase (APX), glutathione reductase (GR), dehydroascorbate reductase (DHAR) and monodehydroascorbate reductase (MDHAR) exhibited significant increases in both content and activity, particularly in roots compared with shoots. In addition to biochemical activity, an assessment was conducted to determine whether the expression of genes encoding these enzymes, as well as those involved in the biosynthetic pathways of the related compounds, was affected. The results of this assessment indicated the same trend. The present study underlines the pivotal function of the AsA/GSH cycle in plant defence in the context of elevated Sb(III) exposure, proposing that roots function as a barrier to restrict the translocation of this metalloid to aerial tissues. These findings may contribute to the identification of species best suited to the remediation of Sb-contaminated environments, based on the enzymes and compounds that play central roles in the defensive response described here. Studies such as the present one contribute to advancing our understanding of the mechanisms by which plants can enhance their remediation capacity and safely restore environments contaminated by xenobiotic compounds.

## 1 Introduction

There is an increasing awareness of the repercussions that elevated concentrations of heavy metals and metalloids in agricultural soils can have on plant growth, crop yield, and human health (Mustafa et al., 2023). Among these elements, antimony (Sb) is a trace metalloid that is toxic and non-essential for both plants and animals. It has received increasing attention in recent years (Ortega et al., 2017; Abou Jaoude et al., 2020; Espinosa-Vellarino et al., 2020, Espinosa-Vellarino et al., 2021; Sytar et al., 2021; Tang et al., 2023).

Sb enters the biosphere via both natural geological sources and anthropogenic activities (Vidya et al., 2022), as it is widely used in several industries for the production of flame retardants, plastics, textiles and electronic devices (Nishad and Bhaskarapillai, 2021). The release of Sb into the environment has increased significantly due to industrial expansion and urbanisation, resulting in substantial problems associated with contaminant waste and its challenging recycling (Tang et al., 2022; Safeer et al., 2024). Sb contamination is predicted to rise further in the coming decades due to population growth (Jiang et al., 2021), although global extractable Sb resources are predicted to be completely depleted by around 2050 (Henckens et al., 2016). Given its toxicity to plants, animals and humans, the United States Environmental Protection Agency (USEPA) and the Council of the European Union have designated Sb as a priority contaminant (Bolan et al., 2022).

In soils, elevated Sb concentrations (greater than 36 mg kg^-^¹, as stipulated by the World Health Organization, WHO) have the potential to modify physicochemical characteristics, influencing pH levels, the composition of organic matter. and induce changes in the soil microbiome (Kataoka et al., 2018).

Evidence indicates that plants have the capacity to absorb water-soluble Sb or Sb present in soil through the root system (He et al., 2012), potentially resulting in the accumulation of high concentrations (Vidya et al., 2022; Rajabpoor et al., 2019) that may become toxic (Haider et al., 2024). In plants, Sb has been shown to induce oxidative stress, leading to a number of adverse effects. These include lipid peroxidation, inhibition of photosynthesis, interference with the uptake of essential nutrients (such as calcium (Ca), copper (Cu), potassium (K), zinc (Zn), iron (Fe), manganese (Mn), magnesium (Mg) and sodium (Na)), and reductions in the synthesis of starch, sugars and soluble proteins (Ortega et al., 2017; Zhu et al., 2020; Haider et al., 2024). Consequently, plants exposed to Sb stress exhibit reduced biomass (Vidya et al., 2022; Haider et al., 2024).

Sb is predominantly present in ecosystems as the pentavalent ion Sb(V) (antimonate) or the trivalent ion Sb(III) (antimonite) (Zhu et al., 2020). Sb(V) has been identified as the most bioavailable form in mining areas (Okkenhaug et al., 2011), whereas Sb(III) has been found to predominate in natural environments (Qi et al., 2021). While both forms are detrimental to living organisms, as they interfere with key enzymatic activities and DNA repair mechanisms, Sb(III) is up to ten times more toxic than Sb(V) owing to its greater mobility and higher ability to cross intracellular membranes (Zhu et al., 2020; Yan et al., 2020; Gan et al., 2023; Tang et al., 2023).

It has been demonstrated that plants exhibit divergent capacities to absorb the two Sb species, a phenomenon attributable to the distinct chemical properties inherent in each. Sb(III) remains neutral in nutrient solutions (Sb(OH)3), whereas Sb(V) is negatively charged (Sb(OH)6-) and is repelled from the root surface, resulting in a lower uptake compared with Sb(III) (Ji et al., 2018). Furthermore, Sb(III) uptake occurs in a manner analogous to that of arsenite (As(III)) via aquaglyceroporins (Maciaszczyk-Dziubinska et al., 2012; Tisarum et al., 2014).

The translocation of Sb from roots to shoots varies depending on the plant species and the Sb form involved (Haider et al., 2024). In general, plants accumulate more Sb(III), particularly in aerial tissues, where Sb(V) levels remain comparatively low (Wei et al., 2015; Barker et al., 2020). However, Shtangeeva et al. (2012) demonstrated that wheat and barley seedlings accumulate higher Sb concentrations in roots, although each species preferentially accumulates a different Sb form: Sb(III) has been identified in wheat, while Sb(V) has been identified in barley. Furthermore, these authors reported a decline in Sb accumulation over time, possibly due to the activation of defence mechanisms such as transporter regulation or the production of root exudates that complex Sb and prevent its uptake. Ji et al. (2017) reported in Lolium perenne that Sb(III) accumulates up to 100-fold more in roots than Sb(V); however, both forms show similar levels in shoots, with a particularly low translocation factor for Sb(III). According to these authors, Sb(III) in roots complexes with thiol groups such as glutathione (GSH) or phytochelatins (PCs) prevent its translocation to shoots and thus contribute to Sb(III) tolerance. In instances where Sb levels exceed thiol availability, an additional tolerance strategy may be employed involving the oxidation of Sb(III) to the less toxic Sb(V). In contrast, Abbasi et al. (2022) reported a negligible role of thiol groups in Sb(V) detoxification and proposed that Sb binding to polygalacturonic acids in the cell wall, with retention in the apoplast, may play a more relevant role.

It has been demonstrated that plants can mitigate the toxicity of Sb through a process known as chelation, in which specific molecules such as PCs or metallothioneins are utilised. These molecules bind to the Sb, thereby sequestering it within the vacuole. In the context of heavy metal detoxification, the role of plants (PCs) is of particular significance. The differential accumulation of metals among plant organs is exhibited in response to a number of factors, including metal concentration, exposure time, growth conditions and species-specific traits. Zhao et al. (2024) reported an increase in PC levels in response to low Cd concentrations, whereas at higher Cd concentrations PC content decreased. Metal-tolerant genotypes have been observed to accumulate higher PC levels in comparison to non-tolerant lines (Srivastava, 2016). However, the correlation between PC content and metal or metalloid concentration is not always evident, suggesting the potential involvement of additional detoxification mechanisms. In circumstances where metal concentrations are minimal, it has been demonstrated that plants may employ alternative defensive strategies in lieu of PC-based detoxification (Faizan et al., 2024). Among the various Sb tolerance mechanisms, an increase in proline content has been documented (Vaculíková et al., 2014; Xue et al., 2015; Tang et al., 2022), which contributes to the maintenance of redox homeostasis and membrane integrity by scavenging excess reactive oxygen species (ROS) (Hayat et al., 2012; Ortega et al., 2017).

The process of phytochelatin biosynthesis is catalysed by phytochelatin synthases (PCS), which are activated in the presence of metal ions. This process continues until the activating metals are chelated, at which point PC biosynthesis is inhibited (Cobbett, 2000). In response to Cd at concentrations that do not alter the overall metabolism of *Arabidopsis thaliana*, increases in PC and reduced glutathione (GSH) content, together with an upregulation of PCS gene expression, have been reported (Moudouma et al., 2012).

The ascorbate-glutathione cycle is a pivotal process in ROS scavenging and metal detoxification. As demonstrated by Wei et al. (2022), in two wheat varieties exposed to zinc (Zn), the levels of reduced ascorbate (AsA) and GSH exhibited an increase or decrease, depending on the Zn concentration. In addition, these researchers documented a dose-dependent response in ascorbate peroxidase (APX) activity and in the content of non-protein thiols and PCs. This concentration-dependent biphasic response is known as hormesis. Moreover, previous studies, including Shaghaleh et al. (2025), have reported that the application of iron oxide nanoparticles (IONPs) or zinc oxide nanoparticles (ZnO-NPs) may mitigate the adverse effects of Cr toxicity in *Triticum aestivum*. This effect has been linked, at least in part, to their interaction with the AsA–GSH cycle, which contributes to the maintenance of high levels of essential antioxidants such as glutathione (GSH) and ascorbate.

It is widely recognised that both GSH and its oxidised form (GSSG) are influenced by Sb(III) and Sb(V) treatments in rice plants (Chen et al., 2024). A plethora of studies have hitherto evaluated the effects of Sb(V) in diverse plant species, including Dittrichia, tomato and sunflower. These studies have reported differential responses in the contents of all ascorbate and glutathione forms, as well as in the enzymes involved in this cycle (Ortega et al., 2017; Espinosa-Vellarino et al., 2020, Espinosa-Vellarino et al., 2021, Espinosa-Vellarino et al., 2024).

In the present study, the effects of Sb(III) on tomato plant growth and on the accumulation of this element in different plant organs were researched. We focused on evaluating the phytoremediation potential of tomato. Although recent studies, such as Jia et al. (2025), have reported that this species is able to hyperaccumulate cadmium (Cd) in the shoots without significantly impairing plant growth, only a limited number of studies have addressed its tolerance and capacity to grow in soils contaminated with heavy metals (HMs). Notable examples include Bolukbasi (2021) and Espinosa-Vellarino et al. (2024), in which tomato cultivars were exposed to Cd and antimony (Sb(V)), respectively, as well as Akpinar and Cansev (2024), who demonstrated that exogenous choline application enhances tomato tolerance to Cd. Furthermore, tomato is a model crop of high agronomic relevance; thus, insights gained from this species may be valuable for understanding metal stress responses in other crops. The study was conducted by exposing these tomato plants to different doses of Sb³^+^ under hydroponic culture, as this system allows precise control of nutrient concentrations, pH, and reduces the variability inherent to soil-based experiments. This provides a more suitable environment for investigating the molecular mechanisms of stress response under well-defined conditions, as described by Chatterjee et al. (2025).

An evaluation was conducted on the involvement of proline and PCs in plant defence against this metalloid. The role of the ascorbate–glutathione cycle was determined by the assessment of the contents of all glutathione forms (total, GSH and GSSG) and ascorbate forms (total, AsA and the oxidised form, DHA). In addition to this, the activities and gene expression of the enzymes involved were also determined. The following enzymes are of particular interest in this study: APX, glutathione reductase (GR), hydroascorbate reductase (DHAR) and monodehydroascorbate reductase (MDHAR).

## 2 Materials and methods

### 2.1 Plant material, growth conditions, and treatments

Seeds of tomato (*Solanum lycopersicum*, L., cv. Tres Cantos) seeds were surface sterilized for 15 min in a 10% sodium hypochlorite solution (40 g L^-1^), rinsed several times with distilled water, and, before germination, were imbibed in distilled water, aerated, and agitated for 2 h at room temperature. After imbibition, the seeds were germinated in a plastic container filled with a sterilized perlite mixture substrate wetted with Hoagland solution, at 27 °C, in darkness for 72 h. After germination, the seedlings were cultivated for 5 days at 27 °C with 85% relative humidity and under constant illumination at a photosynthetic photon flux, density of 350 µmol m^2^ s^-1^1. After 7 days, the plants were grown in hydroponic culture for 7 days in lightweight polypropylene trays and the same environmental conditions as before except for relative humidity of 50%. The plants were treated with a basal nutrient solution consisting of 4 mM KNO_3_, 3 mM Ca(NO_3_)_2_ 4H_2_O, 2 mM MgSO_4_ 7H_2_O, 6 mM KH_2_PO_4_, 1 mM NaH_2_PO_4_ 2H_2_O, 10 µM ZnSO_4_ 7H_2_O, 2 µM MnCl_2_ 4H_2_O, 0.25 µM CuSO_4_ 5H_2_O, 0.1 µM Na_2_MoO_4_ 2H_2_O, 10 µM H_3_BO_3_, and 20 µM NaFeIII-EDTA. For the Sb treatment, the basal solution was supplemented with potassium Sb(III) oxide tartrate trihydrate to final concentrations of 0 µM (CK), 100 µM (24.36 mg L^-1^ Sb) (Sb1), 250 µM (60.89 mg L^-1^ Sb) (Sb2) and 500 µM (121.78 mg L^-1^ Sb) (Sb3). Each culture solution was adjusted to pH 5.8, continuously aerated, and changed every 4 days. The plants were exposed to the Sb for 7 days (their total age at the end of the experiment was 21 days). Data derives from five independent experiments with eight plants per treatment and three technical replicates per measurement, except for mineral content, which was determined using pooled plant material. Plants from each treatment were divided into roots and shoots which were washed with distilled water, dried on filter paper, measured and weighed to obtain the fresh weight (FW). Leaf, stem and root tissues from plants subjected to the different treatments were oven-dried at 70 °C for 48 h to determine dry weight (DW). The dried material was subsequently used for the quantification of Sb(III) and other mineral elements. The % DW was determined: (DW)/FW) x 100.

### 2.2 Leaf area measurement

Photographs of each plant and all their leaves were taken by the DXM 1200C camera (Nikon^®^ Corp., Tokio, Japan). Each photograph was analysed with the free software Fiji Image J 2.3.2. At least 15 different plants were counted and repeated three times. The area was calculated using the polygon and measure tools and all the leaves of each plant were considered for the total area. Photographs were taken in 21-day-old plants.

### 2.3 Quantification of mineral and Sb content

To determine the concentrations of Sb and mineral content in roots and leaves, the samples were maintained at 70 °C for 48 h, and then crushed in a ceramic mortar. The assays were performed by inductively coupled plasma mass spectrometry (ICP-MS) (Lehotai et al., 2012).

### 2.4 Quantification of photosynthetic pigments and determination of photosynthetic efficiency

Disks were taken from fresh adult leaves and homogenized in 80% acetone and centrifuged at 1,500 g for 10 min. The chlorophyll a, chlorophyll b, and carotenoid contents were determined in supernatant spectrophotometrically by measuring A663, A646, and A470. The total chlorophyll and carotenoid contents were calculated in accordance with Wellburn (1994).

The maximum photosynthetic efficiency (FV (variable fluorescence—maximum fluorescence minus minimum fluorescence)/FM (maximum fluorescence)) was determined on fresh leaves of intact plants, before being collected, using a “ChlorophyllFluorometer OS-30p” device (Opti-Sciences). Prior to the excitation, the sampled leaves were kept in darkness for 10 min, then illuminated so as to measure the fluorescence emitted and calculate the FV/FM ratio (Oxborough and Baker, 1997).

### 2.5 Proline quantification

The proline content was determined in accordance with the method of Bates et al. (1973). Briefly, 0.5 g/1.0 g of leaves and roots, respectively, were homogenized in 2.5 mL of 3% sulfosalicylic acid, filtered, centrifuged at 10,000 g for 10 min, and 500 µL of the supernatant was added to a mixture of the same volumes of glacial acetic acid and ninhydrin. The resulting mixture was incubated at 100 °C for 1 h, then placed into ice to stop the reaction. To each reaction tube, 1.5 mL of toluene blue was added, followed by vortexing for 20 s. After 5 min left at rest, the absorbance at 520 nm was measured, expressing the result as µg proline g^-1^ FW.

### 2.6 Quantification of ascorbate–glutathione cycle metabolites and phytochelatins in roots and leaves

To determine the AsA, DHA, GSH, GSSG, and PC contents, roots and leaves (0.5 g mL^-1^) were homogenized in 5% metaphosphoric acid in a porcelain mortar. The homogenate was filtered and centrifuged at 10,000 g for 15 min at 4 °C. The total ascorbate and glutathione assays were done in accordance with De Pinto et al. (1999). The total ascorbate pool was determined in a reaction medium containing the extract, 150 mM phosphate buffer (pH 7.4), and 5 mM EDTA, which was incubated for 15 min in darkness. The result was then complemented with 0.5% NEM (N-ethylmaleimide), 10% TCA, 44% orthophosphoric acid, 4% dipyridyl, and 110 mM FeCl_3_, followed by incubation at 40 °C for 40 min in darkness. The reaction was halted with ice, and the A_525_ was measured. To determine the amount of AsA, 10 mM DTT (DL-dithiothreitol) was added to the reaction medium before incubation in darkness, while 100 µL of water was added to determine the ascorbate pool. The concentration of DHA was estimated from the difference between the total ascorbate pool (AsA + DHA) and AsA.

The total glutathione pool was determined by adding 400 µL of extract to 600 µL of 0.5 mM phosphate buffer (pH 7.5). The reaction medium containing the extract, 0.3 mM NADPH, 150 mM phosphate buffer (pH 7.4), 5 mM EDTA, and 0.6 mM DTNB [5,50-dithiobis-(2-nitrobenzoic acid)] was stirred for 4 min, then 2 U mL^-1^ GR was added and the A_412_ was measured. To determine the GSSG content, the mixture was incubated for 1 h in darkness with 2-vinylpyridine (20 µL) to eliminate GSH, and, to determine the glutathione pool, 20 µL of water was added. The amount of GSH was obtained by the difference between the total pool (GSH + GSSG) and the amount of GSSG.

Phytochelatins were calculated by subtracting nonprotein thiols and GSH. The nonprotein thiols was determined in accordance with De Vos et al. (1992) using Ellman’s reagent (Ellman, 1959), the absorbance at 412 nm was read after 2 min (ϵ = 13,600).

### 2.7 Determination of antioxidant enzymes of the ascorbate/glutathione cycle

To determine the activities of the enzymes involved in the Ascorbate/Glutathione cycle (APX, MDHAR, DHAR, and GR) the roots or leaves (0.5 g L^−1^) were homogenized at 4 °C in 50 mM phosphate buffer, pH 7.5, 0.5 mM PMSF, 1 mM β-mercaptoethanol, 1 g L^−1^ PVPP, and 5 mM AsA for the APX activity. The homogenate was filtered and centrifuged at 39,000× g for 30 min at 4 °C, and the supernatant was used for the enzyme determinations. The APX activity was determined spectrophotometrically by measuring the oxidation of ascorbate at A_290_ for 2 min (ϵ= 2.8 mM^−1^ cm^−1^) (Rao et al., 1996). The reaction mixture contained 0.5 mM ascorbate, 0.2 mM H_2_O_2_, and the enzyme extract, at 25 °C, in 0.1 M phosphate buffer, pH 7.5, and EDTA 0.5 mM, expressing the result as µmol ascorbate min^−1^ mg^−1^ protein. The protein content was determined by the Bradford method (Bradford, 1976). The DHAR activity was determined from the oxidation of DHA at A_265_ for 1 min (ϵ= 14 mM^−1^ cm^−1^) (De Gara et al., 2003) in a medium containing 0.1 M phosphate buffer (pH 6.5), 0.5 mM EDTA, 2.5 mM GSH, 0.5 mM DHA, and the enzyme extract. The DHAR activity is expressed as nmol ascorbate min^−1^ mg^−1^ protein. The MDHAR activity was determined from the oxidation of NADH at A_340_ for 1 min (ϵ= 6.22 mM^−1^ cm^−1^) (Hossain et al., 1984) in a medium containing 50 mM Tris-HCl buffer (pH 7.8), 10 mM AsA, 0.2 mM NADPH, 0.5 units of ascorbate oxidase, and the enzyme extract, expressing the result as µmol NADH min^−1^ mg^−1^ protein. The GR activity was determined at A_340_ from the oxidation of NADPH for 3 min (ϵ= 6.22 mM^−1^ cm^−1^) (De Gara et al., 2003) in a medium containing 0.1 M phosphate buffer (pH 7.5), 0.5 mM EDTA, 0.5 mM GSSG, 0.2 mM NADPH, and the enzyme extract, expressing the result as nmol NADPH min^−1^ mg^−1^ protein.

### 2.8 Gene expression analysis of genes related to the antioxidant response

Leaves from different shoots and lateral roots were collected from plants grown under the various experimental conditions. Plant material was immediately frozen in liquid nitrogen upon harvesting and stored at −80 °C until RNA extraction and purification using the Spectrum™ Plant Total RNA Kit (Sigma-Aldrich^®^, Milwaukee, WI, USA) and RNase-Free DNase (Qiagen^®^, Hilden, Germany, Cat. No. 79254). RNA quantity and quality were assessed with an Eppendorf Biophotometer D30 (Eppendorf^®^, Hamburg, Germany).

RNA integrity was evaluated by electrophoresis on a 1× TBE gel (Invitrogen™, Waltham, MA, USA) containing GelStar™ (Lonza Rockland Inc., Rockland, ME, USA) as the intercalating dye. For each sample, 2.5 µL of RNA was mixed with 10 µL of RNase-free water (Invitrogen™, Waltham, MA, USA) and 2 µL of loading buffer (Invitrogen™, Waltham, MA, USA). Gels were visualised using an Azure Imaging System C200 transilluminator (Azure Biosystems^®^ Inc., Dublin, CA, USA).

Purified RNA (1–2 µg) was reverse transcribed using the High-Capacity cDNA Reverse Transcription Kit (Applied Biosystems^®^, Foster City, CA, USA) in a thermocycler (Eppendorf^®^, Hauppauge, NY, USA) under the following conditions: 25 °C for 10 min, 37 °C for 120 min, and 85 °C for 5 min, yielding single-stranded cDNA.

Real-time amplification was performed using SYBR Green (Thermo Fisher Scientific, Waltham, MA, USA) on a QuantStudio™ 6 Flex Real-Time PCR System (Thermo Fisher Scientific, Waltham, MA, USA). Each target gene was analysed alongside two reference genes, Actin (Act) [Solyc04g011500.2.1] and β-Tubulin (β-Tub) [Solyc04g081490.2.1], employed as constitutive (housekeeping) controls, as recommended for abiotic stress studies (Lovdal and Lillo, 2009) to normalise expression levels.

Relative expression of each target gene was calculated using the 2^-ΔΔCt method.

### 2.9 Statistical analyses

The results obtained from the various biochemical and molecular measurements were first subjected to the Shapiro–Wilk test to assess data normality. Subsequently, a one-way analysis of variance (ANOVA) was performed using RStudio version 4.3.2 (RStudio, Inc., Boston, MA, USA). This analysis was used to determine statistical significance, with significant differences among treatments indicated by different letters (p <no><</no> 0.05). Data are presented as mean values, with error bars representing the standard deviation (SD) of at least 15 replicates derived from five independent experiments. Data visualisation was performed using Microsoft Excel (Microsoft^®^ Corp., Albuquerque, NM, USA).

## 3 Results

### 3.1 Effect of Sb on biomass and mineral nutrient content

Shoot and root length, as well as fresh and dry weight, were determined in plants grown under the different Sb treatments and in control plants.

**Figure 1:**
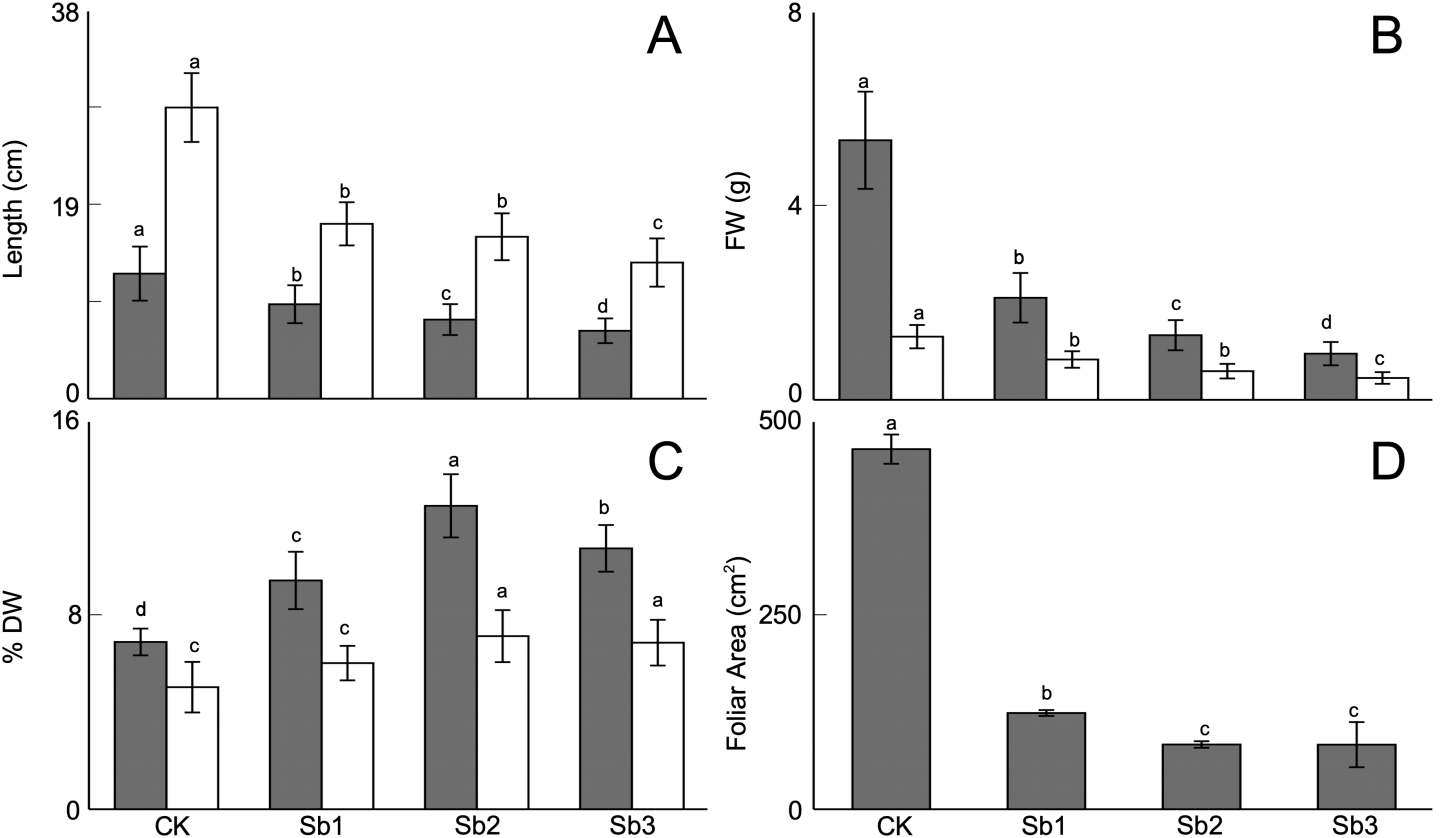
Root length **(A)**, fresh weight (FW) **(B)**, dry weight (DW) **(C)** and leaf area **(D)** of control (CK) samples and plants exposed to different Sb(III) concentrations: Sb1, Sb2 and Sb3. Different letters indicate significant differences according to one-way ANOVA. Data for leaves are shown in black, and data for roots are shown in white.

Antimony exposure significantly affected plant length, causing a marked reduction in the size of both shoots and roots (**Figure 1A**). Shoot length decreased by 24%, 37% and 46% in the Sb1, Sb2 and Sb3 treatments, respectively. Roots exhibited slightly greater reductions, although with a response pattern similar to that of the shoots (39%, 44% and 52% in Sb1, Sb2 and Sb3, respectively). In both organs, growth inhibition was dose-dependent; however, no significant differences were observed between Sb1 and Sb2 in roots. Total leaf area (**Figure 1D**) decreased sharply (75% in Sb1 and 82% in both Sb2 and Sb3), and purplish pigmentation was observed on the abaxial surface of the youngest leaves.

Regarding the effect of Sb on fresh weight (**Figure 1B**), pronounced reductions were observed, which were more marked in shoots and were dose-dependent in all cases.

The percentage of dry weight was determined as an estimate of plant water content (**Figure 1C**). Sb treatment induced an increase in this percentage in both shoots and roots. The greatest increase in shoots was observed in Sb2, whereas no significant differences were detected between Sb2 and Sb3 in roots. These results indicate that Sb exposure leads to a reduction in plant water content.

The results regarding the mineral composition of tomato plants (**Table 1**) demonstrate that exposure to Sb(III) leads to a progressive decline in both macronutrient (Ca, K and P) and micronutrient (Cu, Fe, Mn, Mo, Ni and Zn) contents across all analysed plant organs, including leaves, stems and roots. As shown in **Table 1**, Sb(III) accumulation is dose- and organ-dependent: at high concentrations (Sb3), the metalloid accumulates predominantly in roots; at intermediate doses (Sb2), it is mainly localised in stems; whereas at the lowest concentration (Sb1), Sb(III) preferentially accumulates in leaves, although no substantial differences are observed relative to stems.

**Table 1:** Effect of Sb(III) on Sb content and bioaccumulation, and on the content of other essential elements in the roots, stems and leaves of tomato plants.

Treatment	Sb (mg/Kg)	SbBF	Ca (g/100g)	Cu (mg/Kg)	Fe (mg/Kg)	K (g/100 mg)	Mn (g/Kg)	Mo (g/Kg)	Ni (g/Kg)	P (g/100g)	Zn (g/Kg)
CK Root	80.32	–	1.122	133.89	1021.67	6.004	2028.27	4.92	10.33	1.473	74.15
Sb1Root	59.46	2.44	0.869	28.72	378.06	4.100	560.77	0.15	9.71	0.921	33.96
Sb2Root	236.23	3.88	0.900	9.04	12.25	3.621	279.20	0.18	0.90	0.920	11.53
Sb3 Root	1187.83	9.75	2.743	0.82	3.14	3.427	271.37	<0.01	0.13	1.887	0.60
CK Stem	7.95	–	1.130	6.18	42.53	8.120	94.53	2.38	1.74	0.599	27.64
Sb1 Stem	203.94	8.37	0.943	2.79	51.43	6.530	103.25	0.72	0.81	0.509	9.45
Sb2 Stem	416.55	6.84	0.755	2.37	27.78	4.907	59.55	0.62	1.03	0.445	14.33
Sb3 Stem	272.24	2.24	0.830	2.71	9.85	6.361	82.27	0.63	0.60	0.572	7.23
CK Leaf	15.38	–	2.344	20.63	118.07	3.753	233.65	4.88	1.35	1.163	35.75
Sb1 Leaf	263.95	10.83	2.161	5.71	105.33	2.328	251.82	1.54	1.97	0.902	13.55
Sb2 Leaf	230.68	3.79	1.116	5.30	76.04	1.342	153.21	1.11	1.86	0.495	14.99
Sb3 Leaf	139.54	1.15	1.663	5.56	35.17	2.142	171.67	1.23	0.74	0.749	12.08

### 3.2 Alteration of photosynthetic pigment content and photosynthetic efficiency by exposure to Sb

The content of photosynthetic pigments and photosynthetic efficiency are altered by the presence of SbIII (**Table 2**). With respect to chlorophyll content, Sb induced a decrease in both chlorophyll a and chlorophyll b. Consequently, the chlorophyll a/b ratio increased. In contrast, carotenoid content increased under Sb treatment and, as a result, the carotenoid-to-chlorophyll ratio was also enhanced.

**Table 2:** Effect of Sb(III) on photosynthetic pigment content and photosynthetic efficiency in tomato leaves.

Treatment	Chl a (μg g^-1^ FW)	Chl b (μg g^-1^ FW)	Chl a/b	Carotenoids (μg g^-1^ FW)	Carotenoids /total chl	Fv/Fm
CK	962.9±26.4a	640.9±89.4a	1.48±0.20b	143.9±31.5ab	0.088±0.024c	0.791±0.011 a
Sb1	780.3±143.1b	415.1±73.8b	1.81±0.24a	161.2±23.8a	0.128±0.020b	0.781±0.042 a
Sb2	731.2±128.8b	419.9±65.8b	1.69±0.12a	162.8±12.7a	0.154±0.023a	0.689±0.064 b
Sb3	472.3±103.7c	326.4±76.8c	1.64±0.23ab	142.3±23.4b	0.175±0.028a	0.623±0.155 b

Data are means of at least four independent experiments ± SD. Different letters within each parameter indicate significant differences.

Photosynthetic efficiency was not significantly affected in Sb1; however, in Sb2 and Sb3 it decreased significantly by 13% and 21%, respectively.

### 3.3 Effect of Sb on protein and proline and phytochelatin content

**Figure 2:**
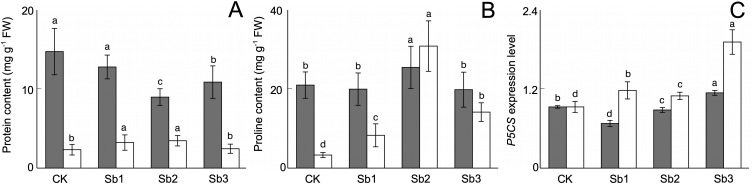
Total protein content **(A)**, proline content **(B)**, and expression of the *P5CS* gene **(C)**, which is involved in proline biosynthesis. Different letters indicate significant differences according to one-way ANOVA. Data for leaves are shown in black, and data for roots are shown in white.

In leaves, Sb caused a reduction in protein content, which was significant in Sb2, with a 39% decrease relative to the control (CK) (**Figure 2A**). In contrast, roots showed a significant increase in protein content at the lower Sb concentrations (39% and 49% increases relative to CK in Sb1 and Sb2, respectively), whereas in Sb3 protein levels were similar to those of CK (**Figure 2A**).

Proline content, an amino acid associated with stress responses (**Figure 2B**), increased in tomato leaves in response to Sb, although this increase was only significant in Sb2 (21%). In Sb1 and Sb3, no significant differences were found relative to CK. In roots, by contrast, proline content increased significantly at all Sb concentrations, particularly in Sb2 (51%, 835% and 339% in Sb1, Sb2 and Sb3, respectively).

With respect to the expression of *Δ¹-pyrroline-5-carboxylate synthetase* (*P5CS*), the key enzyme involved in proline biosynthesis (**Figure 2C**), Sb1 and Sb2 treatments led to a reduction in transcript levels in leaves. In contrast, Sb3 induced a 23% increase in *P5CS* expression. In roots, Sb treatments induced an overall increase in expression, particularly in Sb3 (23%, 18% and 106% in Sb1, Sb2 and Sb3, respectively). Expression levels were generally higher in roots than in leaves, except in control plants, which showed similar levels in both organs.

**Figure 3:**
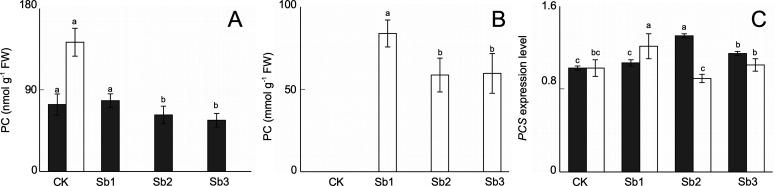
Phytochelatin (PC) content in leaves **(A)** and roots **(B)** of tomato plants exposed to different Sb(III) doses. **(C)** Shows the expression of the *PCS* gene, which encodes a key enzyme involved in PC biosynthesis. Significant differences among treatments are indicated by different letters following one-way ANOVA. Data for leaves are shown in black, and data for roots are shown in white.

The effect of Sb on PC content in leaves (**Figure 3A**) was concentration-dependent. While Sb1 caused a slight increase, Sb2 and Sb3 induced significant decreases of 16% and 23%, respectively. In roots, PC levels in control plants were of the same order of magnitude as in leaves (nmol g^-^¹ FW). However, Sb treatments caused a drastic increase in PC content, reaching mmol g^-^¹ FW values (**Figure 3B**). The highest PC accumulation was observed in Sb1, whereas the increases in Sb2 and Sb3 were approximately 30% lower than in Sb1, although still far higher than in CK.

The expression level of the gene encoding *phytochelatin synthase* (*PCS*) (**Figure 3C**) was higher in leaf samples from Sb-treated plants, reaching its maximum in Sb2. Significant differences were observed between Sb2 and Sb3 compared with Sb1 and CK, which showed similar values. In roots, *PCS* expression exhibited an irregular pattern: expression increased in Sb1 and Sb3 relative to CK, whereas in Sb2 it was reduced.

### 3.4 Effect of Sb on glutathione and ascorbate content

**Figure 4:**
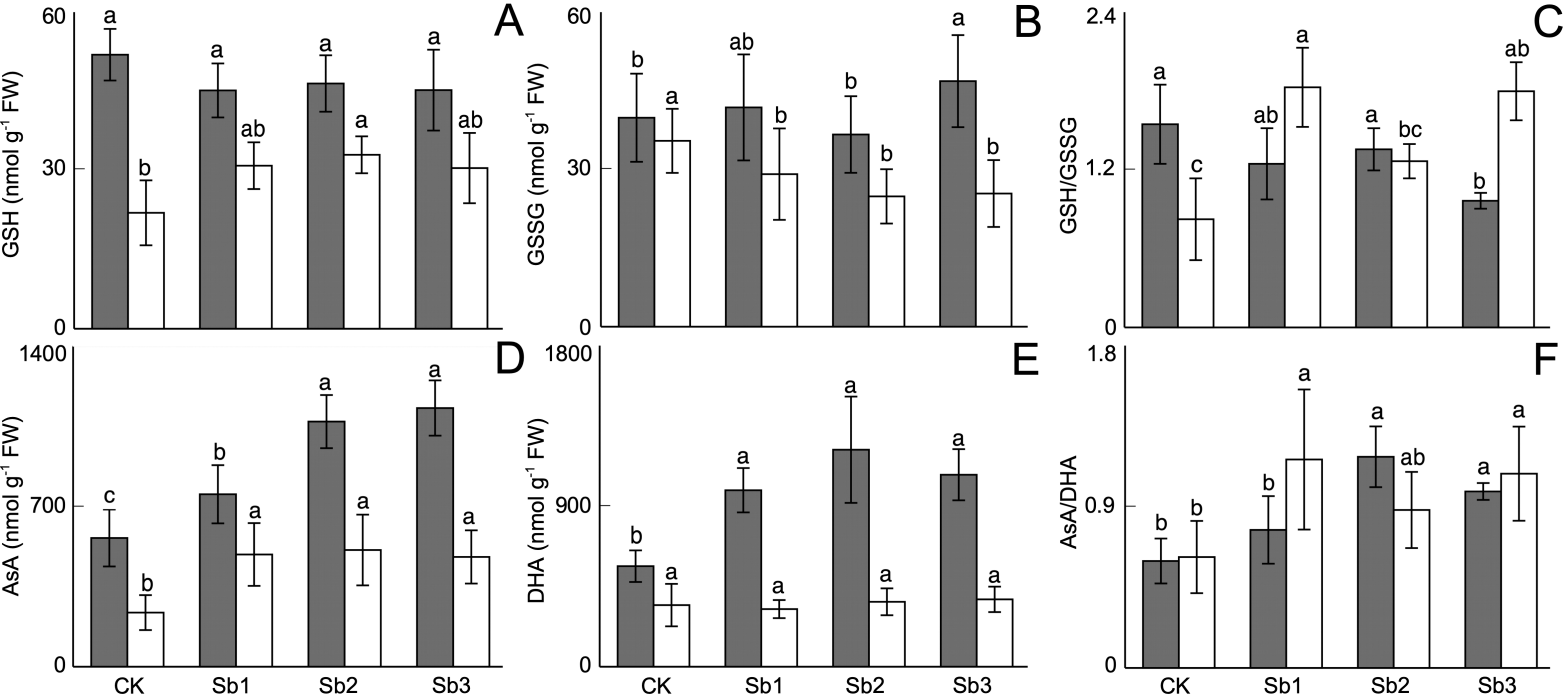
Effect of Sb(III) on GSH **(A)**, GSSG **(B)**, AsA **(D)** and DHA **(E)** contents, as well as on the GSH/GSSG **(C)** and AsA/DHA **(F)** ratios. Significant differences among Sb(III) treatments are indicated by different letters. Data for leaves are shown in black, and data for roots are shown in white.

Reduced and oxidised forms of glutathione and ascorbate, as well as their total pools, were quantified. Regarding total glutathione (**Figure 4A**), no effect was observed in either leaves or roots in Sb1. At higher Sb concentrations, total glutathione significantly decreased in leaves (14% and 21% in Sb2 and Sb3, respectively, with no significant differences between them). In roots, Sb2 caused no substantial change, whereas Sb3 induced a significant decrease of 28%.

GSSG showed contrasting patterns in leaves and roots (**Figure 4B**). In leaves, Sb had no significant effect, whereas in roots GSH increased at all concentrations (41%, 50% and 39% in Sb1, Sb2 and Sb3, respectively). As for GSSG (**Figure 4C**), a significant increase was only observed in leaves in Sb3 (18% relative to CK). In contrast, GSSG decreased in roots at all Sb concentrations (18%, 30% and 28% in Sb1, Sb2 and Sb3, respectively).

Total ascorbate (**Figure 4D**) increased in leaves in a dose-dependent manner, reaching a 106% increase in Sb3. In roots, ascorbate also increased, although to a lesser extent, with the greatest rise observed in Sb2 (41%). AsA (**Figure 4D**) followed a similar trend, with the greatest increase in leaves at the highest Sb concentration (101%). In roots, Sb treatment caused an approximately twofold increase in AsA at all concentrations. DHA (**Figure 4E**) increased in leaves under Sb treatment, with no significant differences among Sb treatments, whereas in roots DHA content was not significantly affected. The ratio between reduced and oxidised forms of these antioxidants reflects the cellular redox state. The GSH/GSSG ratio (**Figure 4C**) decreased in leaves under Sb treatment, significantly in Sb3 (38%). By contrast, this ratio increased in roots, with the highest values recorded in Sb1 (122%) and Sb3 (118%). Sb exposure also increased the AsA/DHA ratio (**Figure 4F**) in both organs: the greatest increase in leaves (97%) was observed in Sb2, whereas in roots the largest increase (88%) occurred in Sb1.

### 3.5 Response of antioxidant enzymes to Sb exposure

The effect of the different Sb doses on both the activity and expression of the enzymes of the AsA–GSH cycle (APX, GR, DHAR and MDHAR) was analysed.

**Figure 5:**
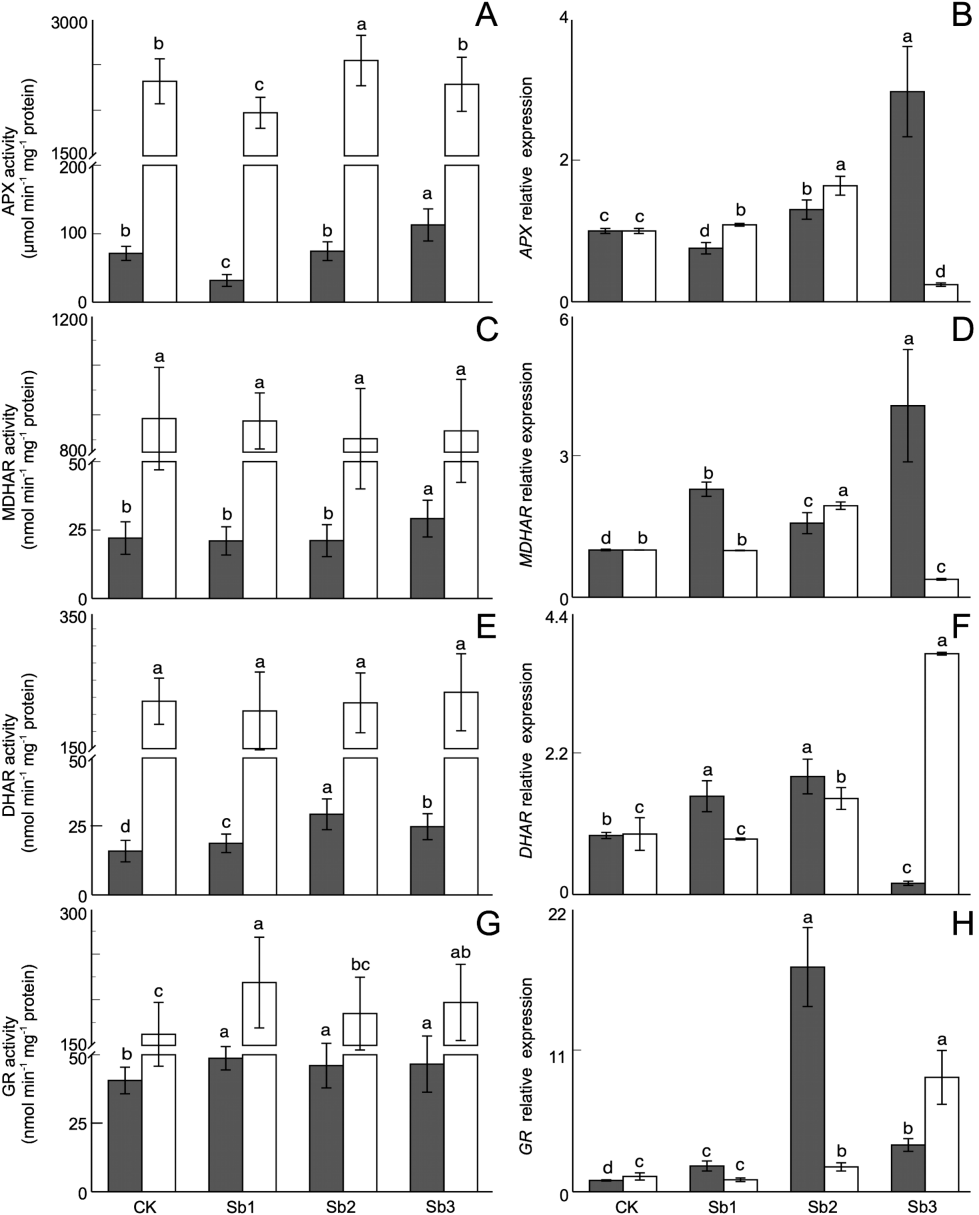
Enzymatic activities of APX **(A)**, MDHAR **(C)**, DHAR **(E)** and GR **(G)** in response to Sb(III)-induced stress, and of the expression of the genes encoding APX **(B)**, MDHAR **(D)**, DHAR **(F)** and GR **(H)**. Significant differences are indicated by different letters following one-way ANOVA. Data for leaves are shown in black, and data for roots are shown in white.

**Figure 5A** shows the effect of Sb on APX activity. Basal APX activity was approximately 30-fold higher in roots than in leaves. In leaves, APX activity was inhibited in Sb1 (55%), recovered to control levels in Sb2, and increased in Sb3 (53%). In roots, Sb1 caused a slight but significant inhibition (15%), whereas in Sb2 APX activity increased significantly above control values (10%), and in Sb3 it returned to CK levels.

Quantification of *APX* expression (**Figure 5B**) showed that Sb3 produced the highest transcript accumulation in leaves (197% increase relative to CK) and the lowest in roots. In Sb2, expression levels were higher than in CK in both organs. In Sb1, expression was higher in roots (above CK) but decreased in leaves.

As observed for APX, GR activity was higher in roots than in leaves (approximately fourfold; **Figure 5C**). Sb induced increases in GR activity in both organs. In leaves, GR activity increased similarly at all Sb concentrations (16%), whereas in roots the highest activation was observed in Sb1 (35%).

With respect to *GR* expression (**Figure 5D**), Sb increased transcript levels in leaves (2.3-, 20- and 4-fold in Sb1, Sb2 and Sb3, respectively). In roots, expression was also induced, but only at the highest concentrations (1.6-fold in Sb2 and 7.5-fold in Sb3). Sb3 showed higher *GR* expression in roots than in leaves, whereas in Sb1 and Sb2 the increase was greater in leaves.

DHAR activity was higher in roots than in leaves (**Figure 5E**). In leaves, Sb significantly increased DHAR activity at all concentrations, particularly in Sb2 (18%, 84% and 56% in Sb1, Sb2 and Sb3, respectively). In roots, DHAR activity was scarcely affected by Sb.

Regarding *DHAR* expression (**Figure 5F**), Sb induced an increase in leaves in Sb1 and Sb2 (66% and 100%, respectively), whereas a marked decrease was observed in Sb3. In roots, Sb1 did not affect *DHAR* expression, but Sb2 and, especially, Sb3 induced significant increases (59% and 298%, respectively). Sb3 caused the strongest modulation of *DHAR* expression in both organs, although in opposite directions.

Roots showed higher MDHAR activity than leaves, with high variability (**Figure 5G**). In leaves, only Sb3 induced an increase in MDHAR activity, whereas in roots no significant effects were observed.

**Figure 5H** shows the effect of Sb on *MDHAR* expression. In leaves, transcript levels increased under all Sb treatments, especially in Sb3. In contrast, in roots *MDHAR* expression increased in Sb2 (94%) but decreased in Sb3 (62%). In Sb3, the expression pattern of *MDHAR* was opposite to that observed for *DHAR*, being highest in leaves and lowest in roots. Only in Sb2 did both organs show increased *MDHAR* expression.

## 4 Discussion

Exposure to Sb(III) causes a reduction in root development in tomato plants, as well as decreases in FW, water content, leaf area (**Figure 1**) and mineral content (**Table 1**). In other words, it affects both the aerial parts and the roots, even though at intermediate doses (Sb1 and Sb2) it accumulates preferentially in leaves and shoots, whereas at higher doses (Sb3) it is retained in the roots. Studies in rice have demonstrated that both Sb(III) and Sb(V) accumulate primarily in roots and shoots, with Sb(III) showing a stronger preferential accumulation in roots, particularly at concentrations exceeding 5 μM (Huang et al., 2024). These observations are in agreement with the present study, where the highest Sb(III) dose promoted root accumulation, while intermediate concentrations favoured translocation to stems or leaves. Previous studies from our group on sunflower and tomato plants exposed to high Sb(V) levels also reported predominant root accumulation, irrespective of dose (Ortega et al., 2017; Espinosa-Vellarino et al., 2020). Taken together, these results suggest that, in tomato plants, both Sb speciation and concentration critically determine its organ-specific accumulation. For another hand, this behaviour has not been observed in other species such as maize, in which it is always accumulated in the shoots (Pan et al., 2011). However, this may be because the amount required for maize to be unable to translocate the metalloid and therefore retain it in the roots has not been reached.

The mineral composition responses of tomato plants subjected to Sb(III) stress are consistent with previous studies. Shtangeeva et al. (2012) reported significant reductions in Ca, K, Na and Cu uptake in wheat plants grown under Sb concentrations of 50–150 mg L^-^¹. Similarly, Feng et al. (2013) observed a marked decrease in the uptake and accumulation of Ca, Mg, Fe, Mn and Zn in rice plants exposed to Sb. However, mineral nutrient alterations under Sb stress appear to be species-specific. For instance, Geng et al. (2020) showed that high Sb exposure in red beet increased Ca concentration while drastically reducing Mn and Zn levels.

Comparable trends have been reported in sunflower plants exposed to Sb(V), where Ortega et al. (2017) observed a dose-dependent decrease in Cu, Fe, Mg and Zn uptake. Collectively, these findings indicate that Sb, regardless of its oxidation state, generally impairs mineral nutrient acquisition and accumulation, although the response of individual elements may vary depending on the plant species. Such nutrient imbalances are frequently associated with reduced biomass production and impaired plant growth under metalloid-induced stress.

At the phenotypic level, in previous studies by our group, it was reported that Sb(V) caused similar symptomatology (Espinosa-Vellarino et al., 2020; Espinosa-Vellarino et al., 2024), although it was not as aggressive; for example, root length was reduced by 11–13% under Sb(V), whereas under Sb(III) this reduction increased to 44–55%. These results are consistent with those described by Zhou et al. (2018) and Chen et al. (2024), who indicate that Sb(III) has a greater inhibitory effect than Sb(V).

The observed reduction in tomato plant growth in the presence of Sb(III) may be attributed to disruptions in photosynthetic metabolism induced by this metalloid. To investigate this, we assessed both photosynthetic pigment content and photosynthetic efficiency. Under stress conditions caused by heavy metal accumulation in the foliar organ, chlorophyll pigments undergo a series of photochemical reactions, including oxidation, reduction, and pheophytinisation (Govindaraju et al., 2010). The chlorophyll a/chlorophyll b ratio is a widely used indicator of foliar stress, as increases in this ratio are often associated with declines in light-harvesting proteins (LHCP) (Mobin and Khan, 2007). In species such as *Empetrum nigrum* (crowberry) exposed to Ni or Cu (Monni et al., 2001), wheat exposed to As (Zengin, 2015), or *Silphium perfoliatum* under Cd or Pb stress (Sumalan et al., 2023), an elevated chlorophyll a/chlorophyll b ratio has been reported, reflecting a shift in the PSII/PSI balance in stressed leaves (Anderson, 1986), which may correspond to enhanced photosynthetic efficiency under stress (Kitajima and Hogan, 2003). Our results (**Table 2**) for intermediate Sb(III) doses align with these findings. However, at higher doses, the ratio declines, although it remains above that of control plants, likely because severe leaf damage at elevated doses compromises or exhausts defensive capacity. Consistently, photosynthetic efficiency decreased with increasing Sb(III) concentrations, likely reflecting impaired electron transport within the thylakoid membranes of foliar tissues, leading to photoinhibition (Zhou et al., 2018). These observations are consistent with reports in tomato and *Dittrichia* (Espinosa-Vellarino et al., 2020, Espinosa-Vellarino et al., 2021). Carotenoids are key pigments in antioxidant defence, contributing not only structurally to photosynthetic reaction centres and light-harvesting complexes, but also protecting the photosynthetic apparatus from oxidative damage by scavenging reactive oxygen species (ROS) (You and Chan, 2015; Houri et al., 2020). Also, under stress caused by heavy metals or metalloids, carotenoid content increases; therefore, carotenoids are considered part of the plant defence system, contributing to antioxidant protection and the mitigation of oxidative damage induced by heavy metal toxicity (Ozavize et al., 2024). While Sb(V) exposure has been reported to decrease carotenoid content in other species, such as sunflower (Ortega et al., 2017) and *Acorus calamus* (Zhou et al., 2018), Zhu et al. (2022) observed no changes in rice exposed to Sb(III) and Sb(V). In our study, Sb(III) treatment led to a significant increase in carotenoid content, although the highest treatment (Sb3) did not elicit a further increase, suggesting that foliar capacity for protective response was compromised under extreme stress.

It is important to note that photosynthesis is closely related to protein synthesis (Tcherkez et al., 2020). Therefore, both total protein content and the content of specific proteins (phytochelatins, PCs) or relevant amino acids (proline) involved in the detoxification processes of xenobiotic compounds were analysed. In general, protein content in tomato leaves decreases following Sb(III) treatment (**Figure 2A**), a phenomenon that has also been observed in rice subjected to Sb(III) and Sb(V) (Chen et al., 2024). This decline may be attributable to either diminished synthesis or augmented protease activity (Faizan et al., 2021). However, in roots, the opposite trend occurs, with an increase in protein content (**Figure 2A**), mainly in Sb1 and Sb2, as also reported by Wang et al. (2024) in Aegilops tauschii exposed to Ni, Cu or Pb (at low doses; at high doses, a decrease in protein content was recorded). Within these proteins, analysis of PC behaviour (see **Figure 3**) revealed a significantly higher amount in the roots of plants subjected to Sb(III), and, within these plants, a higher proportion in roots than in leaves. This phenomenon occurs despite the absence of a significant increase in the expression of the PCS (PC synthesis) enzyme’s coding gene in roots. In fact, only at the Sb1 dose was there higher expression in roots than in leaves. It is well documented that both PCS activity and PC production increase more in roots than in leaves in plants subjected to heavy metal stress (Estrella-Gómez et al., 2009; Moudouma et al., 2012). Consequently, the higher PC content observed in roots compared to leaves may suggest that Sb(III) is being predominantly neutralised in this particular plant segment. The absence of an observed increase in the PCS coding gene, despite reports such as Kısa (2019), which demonstrated increased *PCS* gene expression, may be attributable to the presence of isoforms of this enzyme, a phenomenon that is prevalent in higher plants (Kühnlenz et al., 2014), although not yet identified in tomato plants. It is postulated that these genes do not act redundantly, but rather perform complementary functions with different regulatory mechanisms. This is due to the fact that factors such as the heavy metal or metalloid, its concentration, exposure time and species or cultivar can promote one type of response or another (Xu et al., 2017; Wei et al., 2022; Zhao et al., 2024).

With regard to proline, its increase, especially in roots (**Figure 2B**), may be related to the role of this amino acid in tolerance to Sb-induced stress, as it may be involved in ROS scavenging and metal chelation (Hayat et al., 2012; Ortega et al., 2017). This increase in proline under Sb(III) and Sb(V) has been reported in several studies (Vaculíková et al., 2014; Xue et al., 2015), as well as in response to other heavy metals (Thakral et al., 2024; Soliman and Abdelhameed, 2025; Adamipour et al., 2025). However, these results differ from those described by Espinosa-Vellarino et al. (2020) for Sb(V) in tomato plants, where proline increased only in the aerial part and not in the roots. Consequently, the proline accumulation response exhibited by tomato plants is contingent upon the form of Sb present within their environment, a phenomenon that is further compounded by variations in plant species. In Boehmeria nivea, Sb(III) did not cause any alteration in roots (Lu et al., 2023), and even decreased with dose, while foliar levels increased – i.e. the response shown by the tomato variety analysed in this study under Sb(V) stress. When the expression of a gene encoding a key enzyme in proline synthesis, *P5CS*, was quantified, higher expression was found in leaves than in roots, except in roots of plants subjected to Sb1, which showed higher expression in roots than in leaves. Furthermore, an increase in Sb dose resulted in elevated *P5CS* expression, with the exception of roots treated with Sb2 (**Figure 2C**), a finding that aligns with the quantified proline content. In general, the induction of expression of the gene encoding a rate-limiting enzyme in proline synthesis has been observed in response to abiotic stresses such as those studied here (Adamipour et al., 2025; Renzetti et al., 2025).

The components of the ascorbate–glutathione (AsA–GSH) cycle are essential for reactive oxygen species (ROS) scavenging and for maintaining cellular redox homeostasis, thereby protecting proteins and membranes against stress induced by heavy metals and metalloids (Wei et al., 2022). Indeed, recent studies, such as Ramadan et al. (2025), have reported that tomato plants of a cultivar different from that analysed in the present study (Super Strain B) exhibited marked alterations in key elements of the AsA–GSH cycle, including AsA, GSSG and GSH contents, as well as in the activities of APX, GR and DHAR, under lead (Pb) stress. These findings indicate that the regulation of this cycle plays a crucial role in enhancing plant tolerance to heavy metal exposure. Sb(III) exhibited minimal impact on the content of GSH and GSSG in tomato leaves, as evidenced in **Figures 4A, B**. The content of GSH and GSSG is contingent upon the activity of GR, which is responsible for the production of GSH, and DHAR, which is responsible for the production of GSSG. It was observed that both enzymes exhibited an increase in activity and expression in response to Sb(III) (see **Figures 5E–H**). This resulted in the maintenance of stable GSH levels and an increase in GSSG, consequently leading to a decrease in antioxidant capacity at high Sb(III) concentrations (see **Figure 4C**). The finding that elevated GR activity in leaves did not result in increased GSH levels compared with control plants may be attributable to the translocation of Sb(III) from leaves to roots. This is supported by the observation of enhanced proline and PC content in roots, indicating a more pronounced response to stress in these tissues. This observation lends credence to the hypothesis that the highest GR activity was measured in roots. It has been hypothesised that GSH could be transported via the phloem, a process that would be mediated by specific transporters such as AtOPT6. This hypothesis is supported by the findings of Wongkaew et al. (2018), who observed that AtOPT6 was expressed under stress conditions in plants. In roots, Sb(III) has been shown to cause a decrease in GSSG and an increase in GSH (**Figures 4A, B**), thereby increasing antioxidant capacity (**Figure 4C**). This effect is probably related to increased GR activity and expression (**Figures 5G, H**). With regard to DHAR, the metalloid did not affect its activity, despite increasing expression of its coding gene (see **Figures 5E, F**).

A comparison of these results with those previously obtained in tomato plants exposed to Sb(V) (Espinosa-Vellarino et al., 2020) reveals that the effects of both Sb forms on glutathione content and related enzymes differ. This has also been reported by Lu et al. (2023) and Zhu et al. (2020). Consequently, while Sb(V) in leaves exhibited no substantial impact on glutathione content in any form (with the exception of GSH, which increased at the highest dose), GR activity diminished and its gene expression demonstrated no discernible effect. The activity of DHAR was not influenced by Sb(V). In roots, Sb(V) caused a considerable decrease in all glutathione forms; GR activity and expression were slightly activated, while DHAR activity was inhibited. It was hypothesised that there would be an increase in GSH; however, this was not observed. One potential explanation for this finding is that GSH was involved in metal detoxification or oxidised to counteract oxidative stress, rather than participating in Sb(V) detoxification (Zhu et al., 2020). A comparison of the results obtained in *Helianthus annuus* (sunflower) and *Dittrichia viscosa* under Sb(V) exposure demonstrates that this metalloid form triggered a decrease in all glutathione forms in leaves and roots, and increased DHAR and GR activity (although GR only in leaves) (Ortega et al., 2017; Espinosa-Vellarino et al., 2021). In a recent study, Chen et al. (2024) reported that Sb(III) exhibited a more pronounced effect on foliar GSSG content than Sb(V), which did not alter this compound. However, GR activity remained unaffected by either Sb form. As Liu et al. (2021) describe, an increase in GSH due to Sb(III) has been observed in leaves of Catalpa bungei. The results of the present study are in agreement with those of Moudouma et al. (2012), who reported increased GSH in roots of *A. thaliana* due to Cd toxicity, while no changes were detected in shoots. Chu et al. (2023) described an increase in GSH and a decrease in GSSG in response to Sb(III) in the rice roots. Decreases in GSSG have been reported in roots of two Pteris species (He et al., 2024), one of which showed increased antioxidant capacity, although GSH did not vary in response to Sb(V). In both species, GR activity increased.

Ascorbate content increased in leaves following exposure to different Sb(III) doses (**Figures 4D, E**), which may be related to the activity and expression of enzymes of the AsA–GSH cycle involved in AsA utilisation and regeneration (APX, DHAR and MDHAR; **Figures 5A–F**). APX and MDHAR were only activated at the highest Sb concentration, whereas DHAR was activated at all concentrations, thereby maintaining high levels of AsA and DHA. Greater DHAR activation has the potential to increase AsA, and consequently antioxidant capacity (see **Figure 4F**). In roots, the presence of Sb(III) resulted in an increase in AsA content (**Figure 4D**), thereby enhancing antioxidant capacity (**Figure 4F**). APX, DHAR and MDHAR activities exhibited minimal sensitivity to Sb(III), yet they demonstrated elevated activity levels in all instances (see **Figures 5A, C, E**). A notable exception was APX activity, which exhibited substantial variations in response to Sb(III), exhibiting either increases or decreases, contingent upon the concentration of Sb(III). Gene expression of these enzymes was found to be more affected by Sb(III).

A comparison of these results with those obtained in response to Sb(V) treatment (Espinosa-Vellarino et al., 2020) reveals a divergent pattern. Sb(V) did not alter the ascorbate pool in leaves, although at the highest concentration of AsA, there was an increase in APX activity and expression. The content of DHA and the activity of DHAR remained unaltered. In roots, Sb(V) responses differed from those of Sb(III), with increased DHA and decreased AsA (with reduced antioxidant capacity). This may be due to increased APX expression and activity, which oxidised AsA, while DHAR activity, responsible for reducing DHA to AsA, was low. As posited by other authors, increases in both ascorbate forms, especially DHA, have been observed in rice roots under Sb(III) (50 µM), concomitant with decreased APX activity (Chu et al., 2023). This finding is consistent with the results obtained for Sb1 (100 µM), which also resulted in decreased APX activity in leaves and roots. Lu et al. (2023) reported that Sb(III) increased AsA in roots of Boehmeria nivea, while leaves were unaffected; however, Sb(V) increased AsA in both organs, more markedly in leaves, similar to observations in other plants (Ortega et al., 2017; Espinosa-Vellarino et al., 2021). Increased APX activity in response to Sb(III) and Sb(V) has also been observed by Rajabpoor et al. (2025) in Salvia spinosa. In leaves of ten Catalpa bungei genotypes exposed to elevated Sb(III) concentrations, Liu et al. (2021) demonstrated APX activation. Wei et al. (2022) described that in both leaves and roots of wheat subjected to Zn toxicity, low concentrations caused decreased AsA content associated with increased APX activity, whereas high concentrations caused a clear increase in AsA with decreased APX activity. Thakral et al. (2024) reported that the activity and expression of APX increased in response to Cd in the leaves of Vigna radiata. DHAR activation in response to Sb(V) has been described in both leaves and roots of sunflower and Dittrichia; in the latter, APX activity was also activated (Ortega et al., 2017; Espinosa-Vellarino et al., 2021). In response to thallium (Tl) toxicity, also in Dittrichia, increased DHAR activity was observed in leaves, while roots showed inhibition (Espinosa et al., 2023). The extant literature contains only a paucity of references regarding the effect of metals and metalloids on MDHAR activity. In studies conducted with Dittrichia, MDHAR activity in roots was observed to be higher than in leaves, a finding that has also been documented in the context of tomato plants. In roots, this activity was found to be inhibited by both Sb(V) (Espinosa-Vellarino et al., 2021) and Tl (Espinosa et al., 2023). However, in leaves, the response exhibited variability depending on the heavy metal. Sb(V) was found to inhibit MDHAR, while Tl was found to activate it. The findings of this study demonstrate that the effects of elements and their different forms are not uniform, and that the responses of organs and plants are not either.

The potential discrepancies between the expression of genes encoding the analysed enzymes and their activity levels may be due to both transtranscriptional and post-transcriptional regulatory mechanisms, such as limited translation efficiency, protein turnover, redox-dependent enzyme activation, and direct inhibition of enzyme catalytic sites by heavy metals.

## 5 Conclusions

The impact of Sb(III) stress on tomato plants was found to be detrimental to growth, affecting both the aerial parts and the roots. This decline in development may be attributable to impairment of the photosynthetic process, despite the increase in carotenoids observed in Sb(III)-treated plants, likely as part of the antioxidant defence response. In addition, other plant responses to the metalloid have been observed, including increased synthesis of PCs (occurring in roots) and proline (more evident in roots). Consequently, it can be inferred that PC- and proline-based defences are significantly more pertinent in roots, functioning as the primary defensive barrier against Sb(III).

With regard to the AsA/GSH cycle, the activities of the enzymes involved are generally higher in roots than in leaves. In roots, an increase in GSH and a decrease in GSSG were detected, resulting in increased redox capacity (GSH/GSSG). This increase may be attributed to increased GR activity, with no concomitant increase in DHAR activity. In leaves, total glutathione levels decreased due to Sb(III), although GSH content remained unaffected despite DHAR activation and increased GR activity; this may explain why GSH levels remained stable or were transported to roots. The GSH/GSSG ratio remained stable in Sb1 and Sb2, but decreased in Sb3, thereby reducing the redox capacity. This was primarily due to an increase in GSSG in Sb3. It is hypothesised that GR and DHAR activities may help maintain redox capacity at low Sb(III) concentrations. However, this is not the case at 500 µM, where DHAR activity decreased relative to Sb2 and its expression was drastically reduced. In roots, the presence of Sb(III) resulted in an augmentation of total ascorbate, attributable to an escalation in AsA, while DHA remained unaltered. This phenomenon culminated in an elevated AsA/DHA ratio. This phenomenon does not appear to be attributable to alterations in the activities of AsA–GSH cycle enzymes (APX, DHAR and MDHAR), since although their activities were considerably elevated in comparison to those observed in leaves, they did not manifest a distinct alteration in response to Sb(III). In leaves, the presence of Sb(III) led to an increase in all ascorbate forms, particularly AsA. Consequently, the AsA/DHA ratio increased in Sb2 and Sb3. This phenomenon may be attributed to elevated DHAR and MDHAR activities (the latter exclusively in Sb3), which are known to produce AsA. In contrast, APX, a key player in ascorbate oxidation, exhibited an increase only in Sb3. The absence of correlation between Sb(III) effects on enzyme expression and activity may be attributable to post-translational modifications or alternative regulatory factors.

However, it is important to note that these results cannot be generalised, as responses vary among plants of the same species to the same metalloids or heavy metals, depending on their chemical form and the species analysed. Nevertheless, they do facilitate the identification of the key components involved in responses to such stresses.

Phytoremediation is a sustainable process aimed at removing harmful elements, such as heavy metals and metalloids including antimony (Sb), from natural environments, thereby reducing their toxicity to living organisms (Wang et al., 2024). However, the ability of plants to uptake and accumulate these toxic elements in roots, shoots or other aerial tissues may pose a risk, as they can enter trophic chains and ultimately affect food safety (Qin et al., 2021). For this reason, it is essential to harvest and safely dispose of contaminated plant biomass after the remediation process has been completed (Muthusaravanan et al., 2018). Nevertheless, the efficiency of plant-based remediation remains a challenging process, highlighting the need for further research aimed at improving both its performance and biosafety.

## Data Availability

The original contributions presented in the study are included in the article/**Supplementary Material**, further inquiries can be directed to the corresponding author/s.
