# Supplementary material for: Phytoremediation capacity of tomato against Sb (III) focusing on the AsA/GSH cycle
Source: Front Plant Sci. 2026 May 28;17:1776021. doi: 10.3389/fpls.2026.1776021 (PMC13253285; doi:10.3389/fpls.2026.1776021)
Supplement: Supplementary file 1 [file Supplementaryfile1.pdf]

Supplementary Material

1 Supplementary Table

Primers used for amplification of tomato cDNAs

Gene	ID Gene	Primers (5´-3´)	Size (pb)	Information
**Actin**	Solyc04g011500.2.1	GAAATAGCATAAGATGGCAGACG<br>ATACCCACCATCACACCAGTAT	159	Actin- 41
**β-Tub**	Solyc04g081490.2.1	AACCTCCATTCAGGAGATGTTT<br>TCTGCTGTAGCATCCTGGTATT	180	β-tubulin
**APX**	Solyc06g005150.3	TCTGGTTTTGAGGGACCTTG<br>GCTTTGTCTGATGGCAACTG	113	Ascorbate peroxidase
**DHAR**	Solyc05g054760.2	TGAGCTTGGCTCCAAAACTG<br>CTTCAGCCTTGGTTTTCTGG	144	DHAR 1 (Dehydroascorbate reductase 1)
**GR**	Solyc09g091840.3	AAAGTGTGGAGCAACCAAGG<br>CTGAACGCATGGTCACAAAC	86	Glutathione reductase, cytosolic
**GS**	AF017984	ACATTGCCAAACTACGG<br>ACCTCTGACATCCTCCC	196	glutathione synthetase
**γ-GCS**	AF017983	CTGCATTCTGGGTGGGT<br>CTCGGCTACTTCGTTCA	208	γ-glutamylcysteine synthetase
**MDHAR**	Solyc09g009390.3	AGATCGTTGGTGCATTCCTC<br>AAAACTGATGCCCTCCTGTG	80	Monodehydroascorbate reductase1
**P5CS**	Solyc08g043170	TTTGAGTCACGACCTGATGC<br>GATCTTTTGGCCTCCTTTCC	98	Proline synthase
**PCS**	Solyc09g072620	TGGATGATCTTCCCGTCTTC<br>TCTTGCCTTCGAACTTCAGC	133	Phytochelatin synthase
